# Altmetrics in the evaluation of scholarly impact: a systematic and critical literature review

**DOI:** 10.3389/frma.2025.1693304

**Published:** 2025-12-01

**Authors:** Paloma González, Martha Fors, Ariel Torres

**Affiliations:** 1 Biblioteca, Universidad de Las Américas, Quito, Ecuador; 2 One Health Research Group, Facultad de Ciencias de la Salud, Universidad de Las Américas, Quito, Ecuador; 3 Facultad de Medicina, Universidad Tecnológica Indoamérica, Quito, Ecuador

**Keywords:** Altmetrics, traditional metrics, scholarly impact, research evaluation, hybrid metrics

## Abstract

Altmetrics have emerged as a complementary tool to traditional citation-based metrics in the assessment of scholarly impact. Unlike traditional metrics that primarily capture academic citations over long periods, altmetrics reflect immediate online attention across platforms such as Twitter, blogs, news outlets, and Mendeley. This article critically examines whether altmetrics can serve as a substitute for traditional metrics by exploring their strengths, limitations, disciplinary variations, and correlation with conventional indicators. Through a review of recent empirical studies and theoretical debates, the article argues that while altmetrics offer valuable insights into social impact and engagement, they are not yet mature or standardized enough to fully replace traditional metrics. Instead, a hybrid model that integrates both systems may offer a more holistic and inclusive measure of research influence.

## 1 Introduction

In the realm of scholarly communication, the evaluation of research impact has long relied on traditional metrics such as citation counts, the h index, and journal impact factors. These indicators have become standard tools for assessing academic productivity and influencing funding decisions, academic promotions, and institutional rankings. However, traditional metrics are often criticized for their delayed reflection of impact, their narrow focus on scholarly citations, and their bias toward certain disciplines and publication types (Fire and Guestrin, 2019).

To strengthen the conceptual foundation of this review, it is important to distinguish between academic impact, social impact, and altmetric indicators. Academic impact refers to the measurable influence of research within the scholarly community, typically captured through citations and bibliometric indicators. Social impact encompasses the broader societal, cultural, and policy effects of research beyond academia. In contrast, altmetric indicators serve as proxy measures that reflect online attention and engagement across digital platforms, offering complementary, but not equivalent, insights into how research circulates and resonates within and beyond scientific communities. This refined framework ensures terminological consistency and conceptual coherence throughout the manuscript, establishing a clear foundation for interpreting the evidence presented in the subsequent sections.

In response to these limitations, altmetrics, or alternative metrics, have emerged as a novel means of measuring the broader influence and reach of scholarly work. Introduced in the early 2010s, altmetrics aim to capture the online attention that academic outputs receive through platforms such as Twitter, Facebook, blogs, news articles, policy documents, and reference managers such as Mendeley. Unlike conventional indicators, altmetrics provide real-time data and are often seen as more inclusive of social and public engagement with research (Priem et al., 2010).

Although altmetrics are gaining momentum in the research evaluation landscape, a central question remains: can they truly replace traditional metrics as reliable indicators of scholarly impact? This article examines the purpose, strengths, and limitations of altmetrics in comparison to conventional measures such as citation counts and impact factors. Drawing on recent literature and empirical findings, we critically explore whether altmetrics can function independently in assessing research impact or whether they are best used as a complementary tool. We conclude that while altmetrics provide valuable insights into the visibility and public engagement of research, they are not yet suitable as standalone replacements for traditional metrics, particularly given their methodological variability and sensitivity to disciplinary context.

## 2 Methods

This study follows the principles of a systematic literature review (SLR) to critically examine whether altmetrics can substitute or complement traditional metrics in evaluating scholarly and social impact. The review was designed and reported in accordance with the Preferred Reporting Items for Systematic Reviews and Meta-Analyses (PRISMA) guidelines to ensure transparency and reproducibility.

The review focused on literature addressing key themes including definitions and types of altmetric indicators; their correlation with traditional bibliometric measures such as citations and the h-index; disciplinary differences in altmetric uptake and interpretation; and the validity, reliability, and standardization of altmetric data across platforms like Twitter, Facebook, news outlets, blogs, and reference managers such as Mendeley and Cite ULike.

## 3 Literature search strategy

The primary database used for this review was Scopus, complemented by Web of Science. Searches included publications from 2010 to 2025 and applied to title, abstract, and keywords, using Boolean combinations of keywords such as *altmetrics, alternative metrics, traditional metrics, citation analysis, research impact*, and *academic evaluation*. Search strings were adapted to each database to optimize retrieval. Studies were eligible for inclusion if they presented empirical findings (quantitative or qualitative) or conceptual analyses related to altmetrics and their application in research evaluation, were peer-reviewed journal articles and provided sufficient methodological detail to allow assessment of data sources and indicators.

Exclusion criteria included Publications not focused on the evaluation of research impact (e.g., purely technical descriptions of social media platforms), non-English language articles when no English version was available and duplicates and editorials without substantive analysis.

Data synthesis was conducted thematically, grouping findings into overarching categories such as strengths, limitations, disciplinary trends, and integration models. Particular attention was paid to how altmetrics reflect social engagement and public visibility of research, compared to the more academically insular nature of traditional citation metrics.

Through this synthesis, the paper explores whether altmetrics are sufficiently robust, consistent, and meaningful to stand alone as a replacement for traditional metrics, or whether a complementary or hybrid model might better serve the evolving landscape of research evaluation.

To ensure the inclusion of robust and credible evidence, all studies meeting the initial eligibility criteria were subjected to a methodological quality appraisal using the Critical Appraisal Skills Programme (CASP) checklist for systematic reviews. Each article was independently assessed across the CASP domains (clarity of the research question, appropriateness of methodology, study design, recruitment strategy, data collection, analysis, and relevance of findings). Studies that failed to meet the minimum quality threshold, defined as scoring “yes” on at least 7 of the 10 CASP items or demonstrating adequate methodological rigor in key domains, were excluded from the final synthesis. The CASP appraisal ensured that only studies with sufficient methodological quality were retained for analysis.

**Figure 1:**
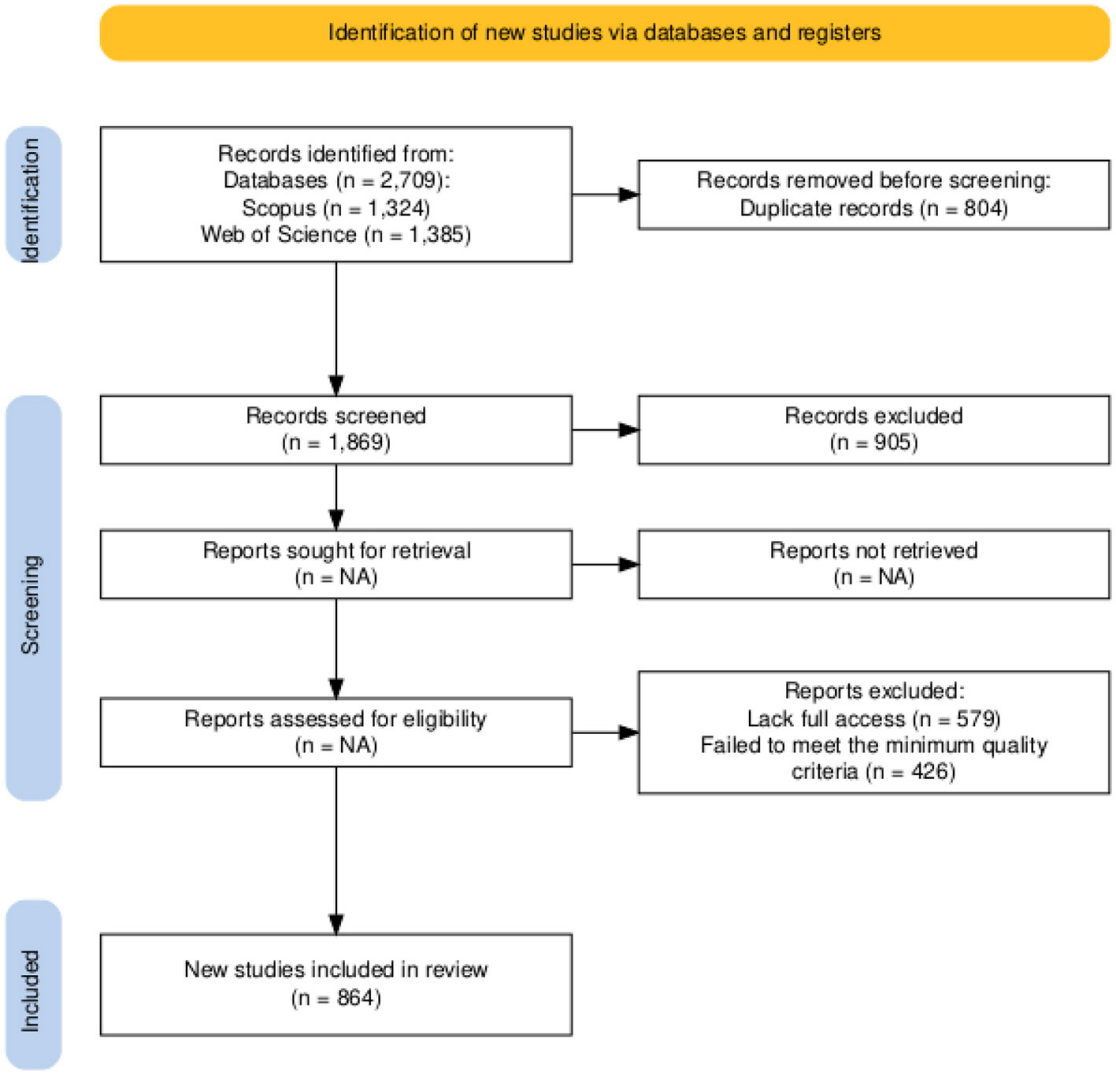
Flow diagram for the selection of studies according to PRISMA guidelines (Haddaway et al., 2022).

A total of 2,709 records were identified through database searches. After removing 840 duplicates, 1,869 articles were screened by title and abstract. Of these, 1,643 were reviewed for eligibility, resulting in 864 studies included in the final synthesis. Figure 1 presents a PRISMA flow diagram summarizing the identification, screening, eligibility, and inclusion stages.

To enhance transparency and traceability, the process of selecting and utilizing the final corpus of 864 (Annex 1) retained titles has been explicitly detailed. After the initial screening and eligibility assessment, these records were subjected to a three-stage analytical process: (1) thematic categorization, in which studies were grouped according to their conceptual focus on altmetrics, academic impact, and evaluative frameworks; (2) methodological synthesis, where the research designs, data sources, and analytical approaches of the included works were systematically reviewed; and (3) critical integration, through which convergences, divergences, and emerging debates were identified to construct the analytical narrative presented in the Results and Discussion sections.

In line with current best practices for research transparency, the authors explicitly disclose the limited and controlled use of generative artificial intelligence (AI) tools during manuscript preparation. These tools were not employed for data synthesis, thematic analysis, or interpretation of results. All processes related to literature screening, coding, categorization, and analytical synthesis were performed manually and independently by the research team.

Generative AI was used exclusively after data extraction and manual synthesis, serving two minor editorial functions: (1) linguistic refinement, including grammar, phrasing, and stylistic adjustments to improve readability; and (2) summarization of author-generated text for conciseness and coherence. No content, interpretation, or critical assessment was generated by AI systems. This clarification ensures full alignment with ethical standards of scholarly writing and maintains the integrity and authenticity of the analytical process.

## 4 Theoretical framework

Traditional metrics refer to established quantitative indicators used to assess the academic impact and productivity of scholarly work. These metrics are typically based on citation analysis and have long been the foundation for evaluating individual researchers, journals, and institutions. Below are the most commonly used traditional metrics:

The impact factor measures the average number of citations received per paper published in a journal during the preceding 2 years. It is a journal-level metric indicating the frequency with which the journal's articles are cited. It is commonly used to assess the prestige of academic journals (Zimba and Gasparyan, 2021).

Journal Citation Reports (JCR) provides the IFs for journals, helping to assess their influence.

**Formula**:


$$\begin{array}{l} \text{Impact Factor =}\\ \frac{\text{Citations in year X to articles published in years X}-\text{1 and X}-\text{2}}{\text{Numbers of articles published in years X}-\text{1 and X}-\text{2}} \end{array}$$

**Example**: If a journal published 100 articles in 2023 and 2024 and these articles were cited 500 times in 2025, the 2025 impact factor would be 5.0.

The h-index is an author-level metric that attempts to measure both the productivity and the citation impact of the publications of a researcher. An h-index of h means that the researcher has h papers that have each been cited at least h times. It is popular for evaluating individual researchers' academic influence, particularly in hiring and promotion decisions (Achugbue and Tella, 2023).

Google Scholar and Scopus automatically display the h-index on researcher profiles, making it a popular metric for evaluating academic impact.

**Example**: A researcher with an h-index of 10 has published at least 10 papers, each of which has been cited at least 10 times.

Citation counts refer to the total number of times a researcher's publications have been cited by other works. This metric is cumulative and increases over time, reflecting the ongoing influence of the researcher's work (Achugbue and Tella, 2023).

**Example**: If a paper has been cited 350 times, its citation count is 350.

Altmetrics, short for alternative metrics, refer to a diverse set of quantitative and qualitative indicators that measure the online attention and engagement a scholarly work receives beyond traditional academic citations. Coined by Priem in 2010 (Priem et al., 2010), altmetrics aims to capture the broader impact of research in the digital age by tracking how academic content is shared, discussed, and used across various web-based platforms.

Unlike traditional metrics, which focus primarily on citations in peer-reviewed literature, altmetrics aggregate data from multiple sources of online activity, providing a more immediate and multifaceted picture of scholarly communication and influence (Wasike, 2021).

Key components of altmetrics include the following:

- Mentions on social media platforms such as Twitter (X), Facebook, LinkedIn, and Reddit reflect the public interest, academic outreach, and discussion surrounding a publication.
- Downloads and views from publisher websites or repositories such as ResearchGate and institutional archives, offering insights into reader engagement and access frequency.
- Blog posts and coverage in online news outlets indicate social relevance and how research findings are disseminated to non-specialist audiences.
- Bookmarks and saves in reference managers and social bookmarking services such as Mendeley, Zotero, and CiteULike, suggesting scholarly interest and intent to use the research for further study or teaching.
- Mentions in policy documents, Wikipedia articles, and patents highlight potential applications of research in public policy, education, or innovation.
- Comments and discussions in open peer review forums or online academic communities such as PubPeer, showing critical engagement and transparency in scholarly discourse (Butler et al., 2017).

Together, these indicators enable a broader and timelier assessment of research impact, particularly in terms of public outreach, interdisciplinary influence, and engagement beyond academia.

To operationalize and visualize alternative impact indicators, several platforms and tools have been developed that aggregate and analyze altmetric data. Among the most widely used tools is Altmetric.com, which enables real-time tracking of online attention to scholarly articles via a distinctive “Altmetric Attention Score”, represented by a colorful donut badge.

The Altmetric Attention Score (AAS) is a composite measure designed to capture the online attention a research output receives across various platforms, including social media, news outlets, and blogs. It is calculated via a weighted algorithm that considers the volume and sources of mentions (Fox et al., 2024).

Another important platform is PlumX Metrics (now part of Elsevier), which organizes metrics into five categories: Usage (clicks, downloads), Captures (bookmarks, saves), Mentions (news, blog posts), Social Media (tweets, shares), and Citations (including Scopus and patent citations). PlumX is often integrated into institutional repositories and Scopus, providing multidimensional visibility for academic outputs (Karmakar et al., 2021).

ImpactStory, a tool developed by OurResearch (formerly ImpactStory.org), focuses on helping researchers tell the story of their broader impact by collecting altmetric data from sources such as Slideshare, GitHub, Twitter, and Mendeley, especially highlighting the openness and accessibility of research outputs and how they are used and cited in various contexts, including policy, practice, and public discourse (Bhabra and Sparks, 2022).

Dimensions, created by Digital Science, integrate traditional metrics (citations) with altmetrics and funding data. It provides a comprehensive research analytics environment that allows users to track both the scholarly and the social influence of publications, datasets, clinical trials, and patents in one interface (Herzog et al., 2020).

However, the data contained in these databases—and the ways in which they are defined, collected, and structured, present significant methodological and conceptual limitations that require a highly critical approach. The proprietary and opaque nature of many altmetric platforms, such as Altmetric.com or PlumX, restricts transparency in how attention scores are calculated and weighted (Haustein, 2016; Ortega, 2020).

## 5 Comparison between altmetrics and traditional metrics

The comparison between altmetrics and traditional metrics reveals distinct strengths and limitations across several dimensions (Table 1). In terms of temporal coverage, altmetrics provide immediate feedback through online platforms such as social media, blogs, and news outlets, capturing early attention and engagement shortly after publication (Sharp et al., 2024; Sonmez and Golbasi, 2024). Traditional metrics, in contrast, reflect long-term academic recognition, as citation counts accumulate over months or years, offering a more stable measure of sustained scholarly influence. Regarding audience and reach, traditional indicators are confined to academic circles, whereas altmetrics extend the scope of impact by incorporating interactions from broader audiences, including the general public, journalists, and policymakers (Hussain et al., 2025). However, questions of validity and reliability persist. While traditional metrics are well-established and standardized, altmetrics remain inconsistent and show variable correlations with citation counts, suggesting they capture different dimensions of impact (Ayoub et al., 2023). Another critical issue is susceptibility to manipulation: altmetrics are particularly vulnerable due to the ease of generating online attention, whereas traditional metrics, though not immune to self-citations or citation cartels, are generally less prone to artificial inflation (Peres et al., 2022). Finally, both systems exhibit disciplinary bias, altmetrics tend to favor fields with strong digital visibility and public engagement, such as health and social sciences, while traditional metrics privilege established disciplines with dense citation networks, often underrepresenting emerging or interdisciplinary research areas.

**Table 1:** Comparison between altmetrics and traditional metrics.

**Aspect**	**Altmetrics**	**Traditional metrics**	**References**
Temporal coverage	Provide immediate feedback; mentions on social media, blogs, or news can appear within hours or days	Reflect long-term scholarly recognition; citations and journal impact factors accumulate over months or years	Sharp et al., 2024; Sonmez and Golbasi, 2024
Audience and reach	Broader audience: general public, journalists, policymakers, and practitioners; capture how research resonates beyond academia	Primarily academic audience; impact measured through citations in scholarly articles and journals	Hussain et al., 2025
Validity and reliability	Coverage can be inconsistent; still under scrutiny; variable correlation with traditional metrics; measure different aspects of impact	Well-established and widely accepted; reliable indicators of scholarly influence; may not capture social impact	Ayoub et al., 2023
Susceptibility to manipulation	Can be manipulated through social media promotion or coordinated attention	Less susceptible; self-citation and citation cartels can still influence counts	Peres et al., 2022
Disciplinary bias	More balanced across disciplines with active online presence (e.g., Health Sciences, Social Sciences, Engineering).	Favor fields with higher citation rates and longer publication cycles (e.g., STEM).	Costas et al., 2015; Zhang et al., 2025

Overall, altmetrics complement traditional measures by offering a broader and timelier view of research dissemination, though challenges regarding their validity, comparability, and equity across fields remain.

## 6 Limitations of traditional metrics and altmetrics

Traditional metrics, such as citation counts, impact factors, and h-indexes, have long served as the cornerstone for evaluating scholarly impact. However, these metrics exhibit several notable limitations. First, traditional citation-based metrics tend to reflect long-term impact rather than immediate influence, often requiring months or years before a publication's significance becomes apparent (Lisciandra, 2025). This latency restricts their usefulness in capturing the early dissemination of research findings, especially in fast-moving fields.

Second, traditional metrics focus primarily on academic citations, often disregarding broader social impacts such as public engagement, policy influence, and media attention (Sharp et al., 2024). As a result, they provide a narrow perspective on the true reach and relevance of research outputs.

Third, the reliance on citation counts and journal impact factors has been criticized for fostering a “publish or perish” culture, potentially incentivizing quantity over quality and encouraging citation gaming. Furthermore, these metrics may disadvantage interdisciplinary research, which tends to receive fewer citations because of its cross-cutting nature and diverse audiences (Subaveerapandiyan et al., 2025).

Finally, traditional metrics often suffer from discipline-specific biases. For example, citation practices vary widely across fields, making cross-disciplinary comparisons problematic (Wang and Hu, 2022). Moreover, access to citation databases such as Web of Science or Scopus is limited by subscription, which may exclude research from less well-funded institutions or regions, thus affecting the representativeness of traditional metrics.

Given these limitations, the academic community has increasingly advocated the inclusion of alternative metrics (altmetrics) that can capture a broader, faster, and more diverse range of research impacts.

However, despite offering real-time insights, altmetrics also have notable limitations.

First, their lack of standardization undermines reliability: different platforms use varied data sources and algorithms, making cross-comparisons inconsistent and replication difficult. This inconsistency also affects validity, as it is unclear whether altmetric indicators genuinely reflect academic quality or lasting influence (Gamble et al., 2020; Thelwall, 2020; Shakeel et al., 2022; Silva et al., 2024).

Second, vulnerability to manipulation is a major concern. Scores can be artificially inflated through coordinated social media campaigns or automated bots, raising questions about authenticity. This risk is further exacerbated by the increasing role of AI, which may intensify noise and deliberate gaming (Gamble et al., 2020; Thelwall, 2020).

Third, altmetrics often capture short-term popularity rather than enduring scholarly impact. A paper might go viral for sensational or controversial reasons but hold little academic value over time. Moreover, platform dependency introduces bias: fields that are less active on social media or that use fewer reference managers may appear undervalued (Liu and Huang, 2022).

Coverage across disciplines and document types is highly uneven, with studies showing that a substantial portion of scholarly outputs remain untracked or inconsistently represented (Costas et al., 2015). These inconsistencies are compounded by differences in data collection methods, platform availability, and language bias, all of which challenge the comparability and reproducibility of results across studies (Sugimoto et al., 2017). Consequently, while altmetrics provide valuable insights into the broader digital visibility of research, they should be interpreted as complementary rather than definitive indicators of scholarly impact.

## 7 Correlations between altmetrics and traditional metrics

Across the included studies, the correlation between altmetrics (e.g., Twitter mentions, Mendeley readership, news coverage) and traditional metrics (e.g., citation counts, Journal Impact Factor, h-index) varied considerably. 125 studies reported low-to-moderate correlations, suggesting that altmetrics and citations capture different dimensions of research impact. For example, Mendeley readership tends to align more closely with future citation counts, whereas Twitter and media mentions reflect public attention rather than scholarly influence. This supports the notion that altmetrics measure immediacy and social visibility rather than long-term academic recognition.

Research from Shrivastava and Mahajan (2023); Bayram et al. (2025); Ömür Arça et al. (2025) reveals only weak to moderate correlations between altmetric scores and traditional citation counts, especially for Mendeley readership. For example, the study conducted by Ömür Arça et al. (2025) revealed no significant correlation between the Altmetric Attention Score (AAS) and citations in either Web of Science (WoS) or Google Scholar (*R* = 0.188, *P* < 0.001 and *R* = 0.161, *P* < 0.001, respectively). However, blog mentions showed a weak correlation with citations from both WoS and Google Scholar (*R* = 0.263, *P* < 0.001 and *R* = 0.241, *P* < 0.001, respectively). In contrast, the number of Mendeley readers exhibited a very strong correlation with citations in both WoS and Google Scholar (*R* = 0.889, *P* < 0.001 and *R* = 0.905, *P* < 0.001, respectively).

However, medical research exhibited some of the weakest correlations between altmetrics and citation counts. This trend was particularly evident in clinical and surgical research, where studies often attracted substantial attention through social media platforms, news outlets, and professional forums but did not achieve a proportional increase in academic citations. For instance, research on innovative surgical techniques, minimally invasive procedures, or perioperative outcomes tended to generate considerable public interest and engagement among practitioners and patients on platforms such as Twitter or ResearchGate. Nevertheless, this attention seldom translated into higher citation counts in indexed journals.

This discrepancy indicates that in fields like surgery and plastic surgery, altmetric indicators may primarily capture social visibility, clinical relevance, and professional discourse rather than direct academic influence. These findings suggest that altmetrics in biomedical and surgical sciences often reflect the immediacy of public and professional engagement rather than the slower, cumulative process of scholarly citation. Consequently, in the medical and surgical domains, altmetrics appear to serve as a proxy for knowledge dissemination and translational impact, complementing but not substituting traditional citation-based metrics (Boyd et al., 2020; Shiah et al., 2020; Smartz et al., 2023; Fox et al., 2024).

## 8 Substitution or complementarity?

The debate surrounding the role of altmetrics in research assessment often hinges on whether they should replace traditional metrics or complement them. While altmetrics have brought fresh perspectives to measuring scholarly impact, the consensus in the literature leans toward a complementary rather than substitutive role.

Despite the growing interest in altmetrics, there is currently no empirical consensus or scholarly recommendation advocating for the complete substitution of traditional citation-based metrics. The literature consistently emphasizes that altmetrics and traditional metrics capture different dimensions of research impact, scholarly influence vs. social and online engagement, and should therefore be viewed as complementary rather than mutually exclusive tools (Liu and Huang, 2022; Ayoub et al., 2023; Chingath and Babu, 2023; Shrivastava and Mahajan, 2023; Fox et al., 2024; Sonmez and Golbasi, 2024; Murugappan and Ramalingam, 2025).

While some proponents highlight the limitations of traditional metrics, particularly in terms of delayed recognition and narrow academic focus, no robust studies suggest that altmetrics alone can provide a comprehensive or reliable measure of research quality or influence. The consensus across bibliometric and scientometric research supports integrated or hybrid models, combining qualitative assessments with both traditional and alternative indicators to ensure a more holistic understanding of research impact (Thelwall, 2025). These models recognize that no single metric can capture the full impact of research.

Integrated research impact models aim to provide a comprehensive understanding of scholarly influence by combining traditional bibliometric indicators (e.g., citation counts), altmetrics (e.g., social media mentions, downloads, or Mendeley readership), and qualitative assessments (e.g., expert peer reviews or case studies on policy or social impact). *Leiden Manifesto* (Hicks et al., 2015) outlines ten principles for the responsible use of research metrics, suggesting contextualized, multi-indicator approaches that reflect the diverse pathways through which research can exert influence. Building on this (Moed and Halevi, 2015), propose a multilayered impact assessment framework that emphasizes the integration of direct and indirect metrics to assess academic, social, and technological contributions. Their model supports the triangulation of data sources to reduce bias and enhance interpretive validity.

## 9 Standardization and integrated evaluation frameworks

Further empirical support for integrated models comes from Cruz Rivera et al. (2017), who conducted a systematic review of 24 methodological frameworks for assessing healthcare research impact. Their analysis revealed that most frameworks rely on multiple domains, scientific, social, and policy-based, and recommend combining quantitative and qualitative approaches to account for the complexity of real-world impact. Expanding this work in the health sciences domain (Sarkies et al., 2021), applied a comprehensive framework to evaluate the outcomes of cardiovascular improvement research. Their study illustrated how traditional academic outputs, such as journal publications and citation counts, can be enriched by tracking changes in clinical practice, stakeholder engagement, and health system performance. The findings underscore the value of using integrated assessment models to capture the broader translational impact of research beyond academia.

A notable addition to this movement is the EMPIRE Index, introduced by Pal and Rees (2022). This novel, value-based metric framework was specifically designed to measure the impact of medical publications across three key dimensions: scholarly, social, and social. Unlike single-score systems, the EMPIRE Index breaks down impact into transparent, meaningful domains, enabling stakeholders to interpret how a publication contributes not just to science, but also to practice and public awareness.

Building on this (Rees and Pal, 2024), explored how the impact of medical publications can vary depending on disease area and publication type, using the EMPIRE Index to reveal differences that traditional metrics might overlook. Their findings demonstrate how disease, specific context and content type influence not just who sees a publication, but how it's used and by whom.

Similarly, the *Metric Tide* report (Wilsdon, 2015), commissioned by the UK's Higher Education Funding Council for England (HEFCE), concludes that no single metric can reliably capture research excellence. Instead, it recommends the adoption of responsible, pluralistic evaluation methods that blend expert judgment with a basket of diverse metrics to support fairer and more accurate evaluations. Collectively, these initiatives reflect a growing consensus in the research community: integrated impact models are essential for acknowledging the multifaceted nature of knowledge production and use.

The NISO (National Information Standards Organization) Altmetrics Initiative, launched in 2013, established key recommendations for defining, aggregating, and interpreting altmetric indicators to ensure transparency, comparability, and reproducibility across platforms. It emphasized the need for data provenance, clear methodological documentation, and the differentiation between indicators of attention (e.g., tweets, news mentions) and indicators of engagement or impact. By promoting these standards, NISO sought to enhance the credibility and interoperability of altmetrics as complementary tools for research evaluation (Carpenter, 2014; Carpenter et al., 2016; Lagace, 2016; Carpenter and Lagace, 2017).

Similarly, the Snowball Metrics Framework, developed collaboratively by universities and research institutions (including Elsevier and several global partners), proposes a standardized methodology for evaluating research performance by combining traditional bibliometrics with emerging alternative indicators. Snowball Metrics emphasize institutionally verified, cross-disciplinary data that integrate publication outputs, collaboration networks, social engagement, and digital visibility. Within this framework, altmetrics are considered part of a broader ecosystem of indicators that reflect the multi-dimensional nature of impact—academic, social, and economic.

Incorporating these frameworks into evaluative practices could mitigate the conceptual fragmentation observed in current studies and provide a more coherent, evidence-based understanding of how altmetrics relate to traditional measures of scholarly influence. Such integration would support a balanced approach to assessing research that values both scientific quality and social relevance (Clements et al., 2017; Snowball Metrics, no date).

## 10 Conclusions

This study highlights the complex and evolving relationship between altmetrics and traditional research metrics. Our findings suggest that while altmetrics offer valuable insights into the broader social and online engagement of academic work, they do not provide a complete substitute for traditional citation-based metrics. Rather, they serve as a complementary tool that can enrich our understanding of research impact, especially in the early stages of dissemination and across diverse audiences.

For researchers, altmetrics offer real-time indicators of visibility and engagement, enabling broader dissemination and fostering collaboration beyond institutional boundaries. Publishers can leverage altmetric data to identify emerging areas of interest, improve content strategies, and enhance audience engagement. For institutions and research evaluators, integrating altmetrics with traditional bibliometrics provides a more comprehensive, multidimensional assessment of research influence—balancing academic excellence with social relevance.

Future studies should focus on developing standardized, integrated frameworks—such as those inspired by the NISO Altmetrics Initiative and the Snowball Metrics Framework—that combine quantitative indicators with qualitative insights. Additionally, longitudinal and domain-specific analyses, especially in disciplines such as medicine and surgery where correlations remain weak, are essential to understanding the true predictive and evaluative value of altmetrics. Addressing persistent challenges related to data quality, manipulation, and transparency will be fundamental to ensuring the reliability and legitimacy of altmetrics in research evaluation systems.
